# Supplementary material for: What Are the Key Factors for the Detection of Peptides Using Mass Spectrometry on Boron-Doped Diamond Surfaces?
Source: Nanomaterials (Basel). 2024 Jul 24;14(15):1241. doi: 10.3390/nano14151241 (PMC11314266; doi:10.3390/nano14151241)
Supplement: Supplementary file 1 [file nanomaterials-14-01241-s001.zip › nanomaterials-3095581-supplementary.pdf]

Supporting Information

What Are the Key Factors for the Detection of Peptides Using Mass Spectrometry on Boron-Doped Diamond Surfaces?

Juvissan Aguedo [1], Marian Vojs [2], Martin Vrška [2], Marek Nemcovic [3], Zuzana Pakanova [3], Katerina Aubrechtova Dragounova [4], Oleksandr Romanyuk [4], Alexander Kromka [4], Marian Varga [5], Michal Hatala [6], Marian Marton [2] and Jan Tkac [1,*]

[1] Institute of Chemistry, Slovak Academy of Sciences, 841 04 Bratislava, Slovakia; aguedo-ariza@savba.sk (J.A.), jan.tkac@savba.sk (J.T.)
[2] Institute of Electronics and Photonics, Faculty of Electrical Engineering and Information Technology,
Slovak University of Technology, 841 04 Bratislava, Slovakia; marian.vojs@gmail.com (M.Vo.);
martin.vrska@stuba.sk (M.Vr.); majko.marton@gmail.com (M.M.)
[3] Centre of Excellence for Glycomic, Slovak Academy of Sciences, 841 04 Bratislava, Slovakia; marek.nemcovic@savba.sk (M.N.); zuzana.pakanova@savba.sk (Z.P.)
[4] FZU - Institute of Physics, Czech Academy of Sciences, Cukrovarnická 10, 162 00 Prague, Czech Republic; dragounova@fzu.cz (K.A.D.); romanyuk@fzu.cz (O.R.); kromka@fzu.cz (A.K.)
[5] Institute of Electrical Engineering, Slovak Academy of Sciences, 841 04 Bratislava, Slovakia; marian.varga@savba.sk (M.V.)
[6] Department of Graphic Arts Technology and Applied Photochemistry, Faculty of Chemical and Food Technology, Slovak University of Technology, 812 37 Bratislava, Slovakia; michal.hatala@stuba.sk (M.H.)
* Correspondence: jan.tkac@savba.sk; Tel.: +421-2-5941-0263

Table S1. Class of amino acids present in the peptides expressed in %.

Peptide N°	Sequence	Hydro-phobic #	Hydro-philic #	Basic #	Acidic #	Hydrophobicity¶ kcal/mol
Brad	RPPGFSP	57.2	28.6	14.3	0	10.03
Ang II	DRVYIHPF	62.5	0	25.0	12.5	11.82
Ang I	DRVYIHPFHL	60.0	0	30.0	10.0	12.90
Sub P	RPKPQQFFGLM	54.5	27.3	18.2	0	10.14
Bomb	OQRLGNQWAVGHLM	42.9	35.7	21.4	0	11.51
Ren S	DRVYIHPFHLVIHN	57.1	7.1	28.6	7.1	14.50
AC1	SYSMEHFRWGKPVGKKR	35.3	23.5	35.3	5.9	23.60
AC18	RPVKVYPNGAEDESAEAFPLEF	54.6	13.7	9.1	22.7	28.75
Som	SANSNPAMAPRERKAGCKNFFWKTFTSC	39.3	39.3	17.9	3.6	23.48

# class of amino acids expressed in % was calculated using an on-line tool: https://www.protpi.ch/Calculator/ProteinTool#AminoAcidComposition;
¶ Hydrophobicity in Kcal/mol was calculated using on-line tool: https://pepdraw.com/

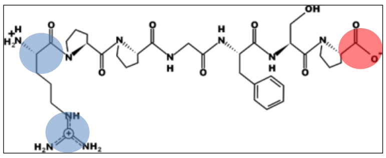

Brad

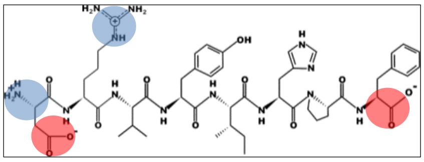

Ang II

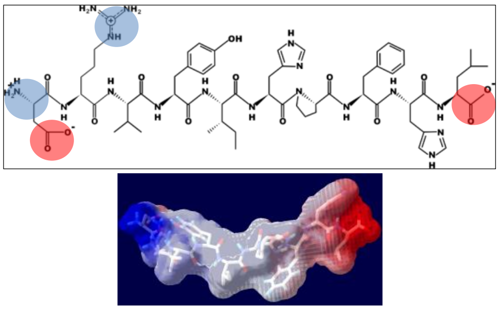

Ang I

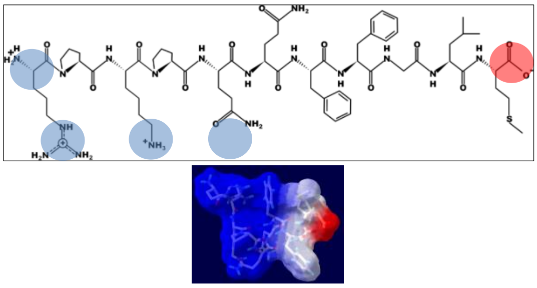

Sub P

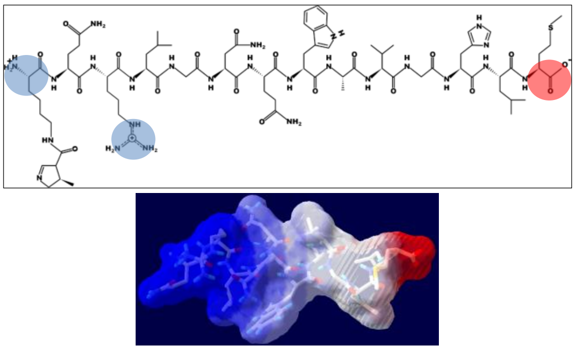

Bomb

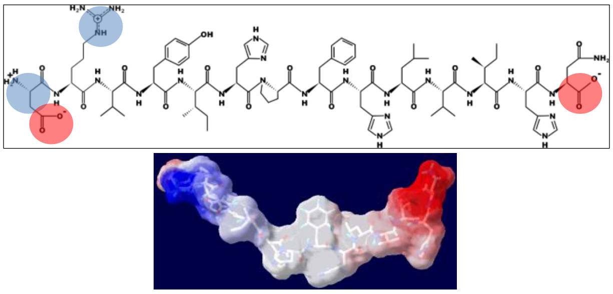

Ren S

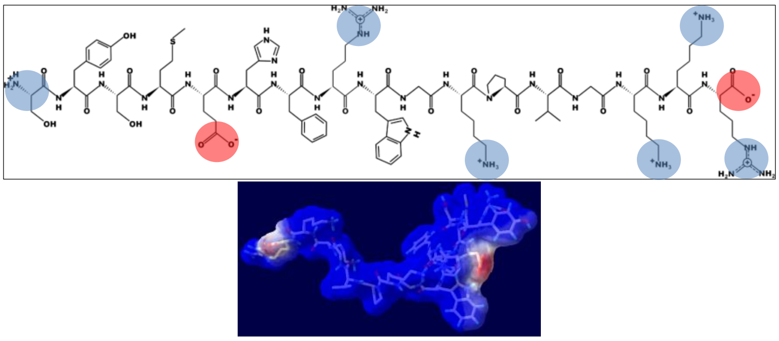

AC 1

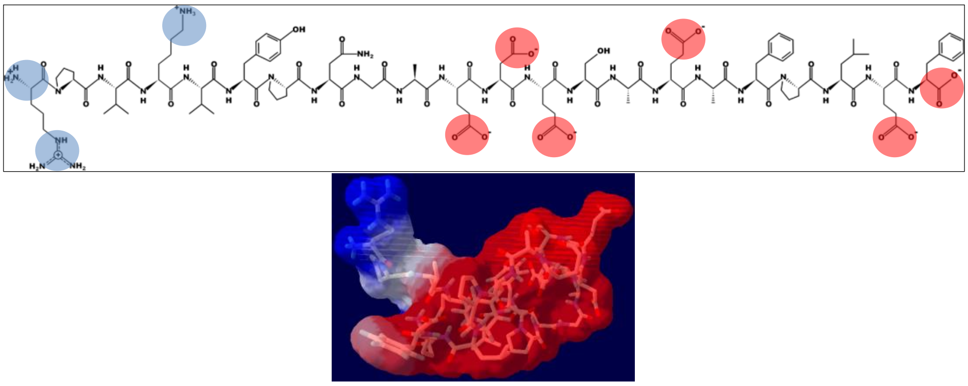

AC 18

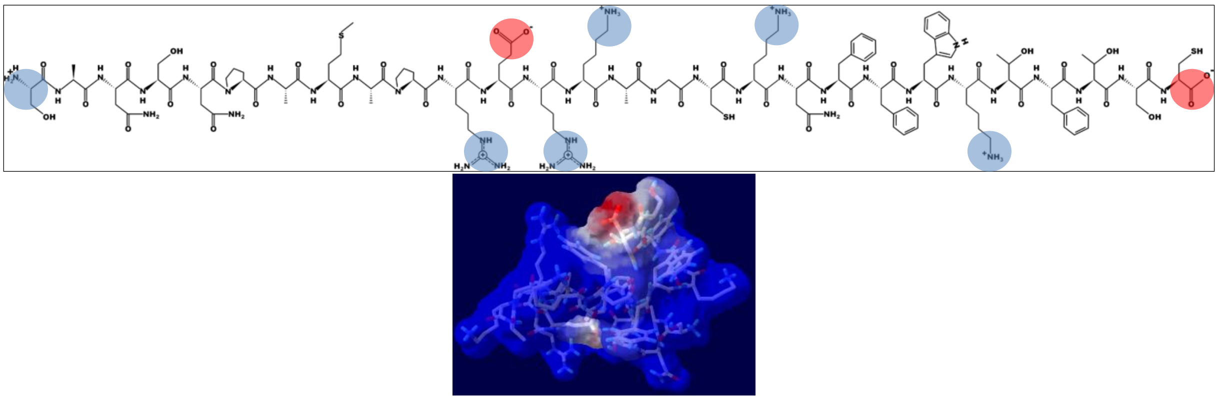

Som

Figure S1: The amino acid composition of peptides (2D structure) with 3D structure shown, as well. 3D structure was generated from PDB file, which was computed by the on-line tool I-TASSER through the web page of Yang Zhang´s research group: https://zhanggroup.org/I-TASSER/. The 3D images of peptides were visualised using Deep View/Swiss-PdbViewer software showing peptide backbone and the surface of the peptide using electrostatic potential values. Red features indicate the presence of a negative electrostatic potential, blue features a positive electrostatic potential and white features hydrophobic patches on the surface of peptides. *Brad* and *Ang II* are too short peptides for which generation of a pdb file was not possible.

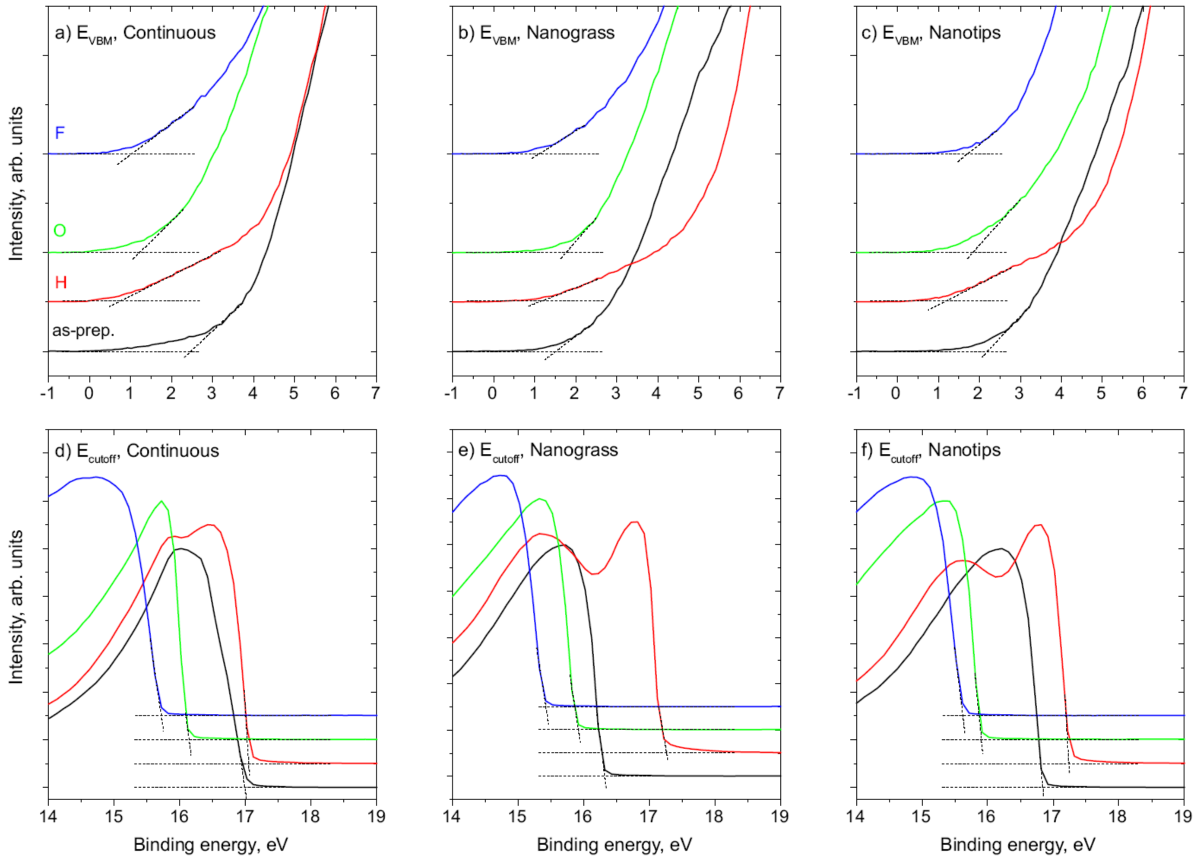

Figure S2: UPS **(a-c)** VBM and **(d-f)** cutoff spectra of continuous, nanograss, and nanotips samples with as-grown (black), -H (red), -O (green), and -F (blue) terminations. The measured values of extrapolated spectra edges are included in **Tab. 3**.
